# Beyond Traditional: Clearing the Roadblocks to Advancement in Academic Medicine

**DOI:** 10.5334/pme.1681

**Published:** 2025-05-14

**Authors:** Pilar Ortega, Mara L. Becker, Teresa M. Chan, Kimberly D. Manning

**Affiliations:** 1 Departments of Medical Education and Emergency Medicine, University of Illinois College of Medicine, Chicago, IL, USA; 2 Faculty at the Duke University School of Medicine and a faculty leader at the Duke Clinical Research Institute, Durham, NC, USA; 3 Toronto Metropolitan University, Toronto, Ontario, Canada; 4 Division of Emergency Medicine and Division of Education & Innovation, Department of Medicine, McMaster University, Hamilton, Ontario, Canada; 5 McMaster Education Research, Innovation, and Theory Program, Faculty of Health Sciences, McMaster University, Hamilton, Ontario, Canada; 6 Emory University School of Medicine, Department of Medicine, Atlanta, GA, USA; 7 RYSE Council on Diversity, Equity, and Inclusion, Atlanta, GA, USA; 8 Small Group Advisor, Atlanta, GA, USA

## Abstract

In academic medicine, the label of nontraditional is often used to refer to scholars whose outputs or journeys differ from what is considered normative. Those who do not sufficiently align with traditional expectations may be at risk of being excluded from fully participating or achieving advancement in academic medicine, an experience that disproportionately affects groups who have been historically marginalized and underrepresented in medicine. In this eye opener, the authors use the lenses of their own stories in academic medicine to illustrate some of the traditional roadblocks experienced by these scholars, such as the lack of mentorship, the tendency to overlook or discourage work on nontraditional topics, and difficulty fitting innovative scholarship formats into curricula vitae or promotion packets. To clear the roadblocks, the authors call upon institutional leaders to enhance their processes, support systems, and criteria for learner and faculty academic advancement. Secondly, the authors call upon individuals to consider how they might engage in and frame their scholarly pursuits in a way that their merit can be readily ascertained.

## Introduction

In academic medicine, the label of nontraditional is often used to refer to scholars whose outputs or journeys differ from what is considered normative or common (i.e., traditional). Scholarly outputs can be considered nontraditional when a scholar’s body of work falls outside of the traditional deliverables of peer-reviewed publications, research funding, and national conference presentations [1,2]. Second, outputs can be nontraditional with regards to content, such as pursuing inquiry in fields that have not been historically considered relevant in medicine [3]. Finally, the scholars themselves may be said to be nontraditional if they arrive at academic medicine from atypical paths, such as those outside the most common age group when entering medical school [4] and those who previously completed atypical majors or pursued non-medical careers [5]. Individuals may also be labeled as nontraditional based on personal identity, such as first-generation college students or those who come from low socioeconomic backgrounds [6].

In this eye opener, we use a combined storytelling and scholarly approach to explore how the nontraditional label impacts outcomes for learners and faculty seeking to advance in academic medicine. We conceptualize advancement broadly – ranging from students’ successful completion of each year of medical school training, resident physicians’ advancement to each post-graduate training year, and faculty member recruitment and promotion across faculty ranks. Consider the following faculty member’s experience:

*Her work was meaningful. Every year, her contributions as a midcareer medical educator were lauded by medical students, and her name was the first to come up when a learner wanted guidance. But not just those at her institution; she was known across the country and even in other countries. Her work as a podcast co-host and her prolific and trusted profile on social media platforms brought her well outside of the confines of peer-reviewed journals or oral abstract presentations at meetings and into the ears and pockets of intellectually curious minds everywhere. Without question, she had a substantial national and international reputation – just not the kind that fit comfortably into the rubric of an institutional promotion and tenure committee*.

*“I like the things you’re doing,” her department chair said. “But everything in your portfolio is nontraditional. To get you promoted, we need to help you do more things that count.”*

This story, like the others sprinkled throughout this eye opener, is based on the personal and witnessed lived experiences of one or more of the authors. In this story, the faculty member’s body of work has been labeled as nontraditional by her supervisor and deemed insufficient for advancement. Yet, that which is considered “traditional” is typically considered so because it follows a pattern of how things are or have “normally” been done. Concepts of tradition and normality are themselves subjective, and, according to some scholars, artificial [7] and potentially harmful [8]. By relying on dominant narratives, these concepts may overlook practices that are normative in certain subsets of the population. While useful for statistical benchmarking, such as establishing a mean or average, it is rare for any single individual to correspond precisely to the norm.

Using “traditional” as an inflexible yardstick to which academic medicine measures each individual’s accomplishments may exclude a large number of scholars, particularly those who are most likely to propel innovation in the field or those from marginalized or minoritized groups. The criteria for successful advancement across each stage of academic medicine may feel obscure to some. Medical students and resident physicians who withdraw or are dismissed [9,10] may lack an understanding of the expectations for advancement to the next stage of training [11] or may not know how to achieve those expectations nor what resources (e.g., individualized learning plans) are available [12,13]. At the faculty level, individuals pursuing nontraditional scholarship may not readily identify a path to promotion that aligns with their work or interests, and existing criteria may feel unachievable [14]. This situation may reinforce a sense of “imposter syndrome” that may cause some to give up on promotion opportunities or abandon academic medicine altogether [15,16,17,18,19].

The impact of attrition from medical education and academic medicine is disproportionately felt by groups historically marginalized [9,10,20]. These individuals are also more likely to participate in historically “non-promotable” activities, such as mentorship and community service [21,22]. Medical students underrepresented in medicine report greater intention to work with underserved populations compared to White students [23], a commitment that persists from training to practice across clinical [24], educational [25,26], and research domains [27,28]. Despite the value of these scholarly contributions to reducing disparities and improving population health, underrepresentation worsens along the medical education continuum and progression of academic rank [29]. For example, according to 2022–23 data, while 13.6% of the US population is Black or African American [30], only 10% of medical students [31], 6.5% of residents [32], and 5.2% of active physicians identify as Black [33]. Similarly, compared to 19.1% of the US population [34], 12.3% of medical students [31], 9.7% of residents [32], and 6.3% of active physicians identify as Hispanic/Latinx [33]. Other groups, such as women, may not be underrepresented in medical school admissions [31], but still substantially lag behind in pay [35], promotion rates [29], and leadership positions [36], pointing to obstacles in their path toward academic advancement.

Furthermore, scholars from marginalized backgrounds may be unfamiliar with the unwritten rules that delineate the most efficient path toward advancement in academic medicine – a part of the hidden curriculum [37] that may be evident to individuals who have been immersed in academic settings through familial or community connections, mentorship, or prior exposures but may be obscure to others. Incorporating flexible approaches to recognizing meritorious nontraditional scholarship and supporting the advancement of nontraditional scholars may help academic medicine respond to the evolving needs of the patient population and create inclusive environments within the academic medicine community.

In this eye opener, we use the lenses of our own paths in academic medicine to identify common roadblocks experienced by nontraditional scholars. PO is a multilingual Latina who immigrated to the US as a child and is now an emergency medicine physician, educator, entrepreneur, and researcher focusing on language-concordant health care. MB is a White female pediatric rheumatologist whose career began fully clinical and evolved to encompass research in pediatric clinical pharmacology and clinical trials, local and multi-site quality improvement projects, and several institutional leadership roles. TMC is a Chinese Canadian woman who serves her community as an emergency physician and as founding dean of Canada’s 18th medical school. Her interests include faculty development, academic governance, and interweaving equity within academic structures. KM is a Black American female academic generalist-hospitalist with combined internal medicine and pediatrics training who works clinically and in leadership roles involving undergraduate and graduate medical education and building and maintaining inclusive, diverse environments for learners, faculty, and patients. While this eye opener does not address all potential obstacles to career advancement in academic medicine, we hope it serves as a call to action for institutions and individuals. For each of the roadblocks described, we first call upon institutional leaders to enhance their processes, support systems, and criteria for learner and faculty academic advancement. Secondly, we call upon individuals to consider how they might engage in and frame their scholarly pursuits such that their merit can be readily ascertained.

## The Lack of Mentorship Roadblock

*“Have you still been meeting regularly with your faculty mentor and attending faculty development sessions?” the department chair asked*.

*They nodded, then gave a defeated shrug. “I do both. But no one understands what I’m doing with enough depth to advise me on next steps.”*

Pursuing mentorship for work that is considered nontraditional presents a challenge due to the lack of individuals with aligning expertise. Many early career faculty begin by seeking a mentor with overlapping concordance in as many areas as possible – from gender, race, and ethnicity to specific area of focus. For those with interests that do not follow conventional paths of scholarship and research, this can be a nearly impossible task that ultimately results in missed opportunities for growth, support, and guidance typically afforded by mentorship [38,39]. For instance, faculty members who have built local, regional, or even national reputations on nontraditional platforms, such as podcasts or social media, may not have access to prior examples they can emulate when describing or quantifying their impact [2].

### Recommendations for Institutional Leaders Addressing Gaps in Mentorship

Department leaders can work with learners and faculty to develop an individualized mentorship plan appropriate to their academic goals. Institutions can facilitate mentorship relationships in many forms, including advising, coaching, mentoring groups, peer-mentoring, and mentor-mentee pairings, depending on available resources and the needs of learners and faculty [40,41]. Moreover, institutions can expose students early to diverse academic tracks and faculty engaged in those pathways [42]. Without early exposure to a breadth of options, students may assume or learn based on the hidden curriculum [43,44], that there is a narrow set of academically valued scholarly activities.

Another strategy is to incorporate mentorship conversations as a standard element when providing feedback to learners, such as when program directors meet with residents to discuss their semiannual performance evaluations. In some situations, finding a mentor with similar research priorities or identity concordance (e.g., shared gender, racial, or ethnic experience), may be particularly important to a mentee. Rather than aiming to achieve a mentor-mentee concordant match on all aspects, it may be useful for leaders to have a conversation with mentees about what characteristics to prioritize in a mentorship pairing. Considering multiple characteristics may broaden the pool of potential mentors, which may help alleviate the minority taxation involved in being a senior faculty member who is asked to mentor all learners or junior faculty of a minoritized group [45].

### Recommendations for Scholars Seeking Mentorship

In addition to engaging in collaborative problem-solving with leadership, individuals can consider leveraging social media networks to create a pool of potential collaborators, mentors, and mentees. Social media and digital platforms can spark scholarly ideas, amplify work [46], and bring awareness to a more diverse swath of individuals, some of whom may be potential mentors, allies, or collaborators [47,48]. KDM used social media platforms to share narrative medicine and advocacy ideas, which led to co-authorships, invitations to national presentations and visiting professorships, and ultimately, promotion to the rank of professor. Recent examples of successful social media-driven networks include the American Heart Association #GoRedForWomen campaign to increase awareness of heart disease in women, with over 66 thousand followers [49], and #NationalLatinoPhysicianDay, a grassroots physician-led movement starting in California that is now nationally recognized on October first [50]. Leaders in each of these campaigns leveraged social media platforms to not only build generalized awareness, but also to empower others to become spokespersons for the cause by disseminating evidence-based fact sheets and talking points – a form of group mentorship.

## The Nontraditional Content Roadblock

*She grew up witnessing the struggles of her own family members when receiving language-discordant health care. As a medical student, she was surprised to find little research on this topic and became interested in a career as a clinician-researcher to address gaps*.

*For her residency scholarly project, a requirement for advancement to the next year of training, she shared a proposal on medical Spanish research with her program director. The program director responded, “That’s really cool, but if you want to someday become a funded researcher, you should first focus on something one of our faculty members is already working on.”*

Individuals with personal lived experience and skills in topics traditionally considered “social” (e.g., language, environmental issues, housing, nutrition) rather than “medical” may be explicitly or implicitly disincentivized from pursuing those areas as scholarship [51]. This happens explicitly when a supervisor tells a learner or junior faculty member that the topic of interest is not appropriate as a scholarly pursuit, irrespective of methodological rigor. For example, a scholar may be dissuaded from pursuing scholarly work on language yet simultaneously overburdened with requests to use their personal time to fill in gaps in curricula through uncompensated extracurricular activities (e.g., leading or supporting the medical Spanish club [52,53]) that are not officially recognized by the institution and may not “count” toward academic advancement. While these “subtle acts of exclusion” [54] are not intended to be personally demeaning, their impact may be substantial: When scholars’ lived experiences and identity align with their area of inquiry, they are uniquely positioned to develop well-informed, innovative research questions and interpret data in the context of those experiences and contribute to the field in ways that others might miss [55,56]. When the topics that provide meaning in medicine are devalued or deemed inadequate, these individuals may experience a greater degree of burnout compared to individuals on a more traditional path [20]. Some may feel pressured into pursuing areas of scholarship to which they do not feel a personal connection; the cognitive dissonance involved in separating one’s passions and personal identity from one’s professional work may also take a toll [57].

### Recommendations for Institutional Leaders on Nontraditional Scholarly Topics

Institutional leaders should consider how learners and faculty are being advised regarding their areas of academic engagement. For example, if institutions wish to encourage interdisciplinary work across medicine and the humanities, this should be described as an example of scholarly inquiry that would be valued [58]. Institutions may consider fostering spaces that allow individuals from different departments, health professions, and disciplines within or across institutions to meet and explore opportunities for collaborative scholarship.

Program and department leaders could also provide guidance on how to effectively frame contributions as academic assets on a school/job application, interview, or promotion packet. For example, some scholars recommend describing language proficiency as a professional skill as part of a frameshift away from the historically deficit-based approach to individuals who speak non-English languages [59]. Similarly, community service activities can be framed as professional contributions [60]. Institutions should ensure that faculty members can easily access promotion guidelines and that the guidelines include feasible ways to report mentorship and committee service contributions and impact.

### Recommendations for Scholars Pursuing Nontraditional Topics of Inquiry

Having a group of scholars with a shared nontraditional interest can be helpful by creating a network of collaborators who may share and refine ideas together, engage in multi-site studies, and provide critical review or peer mentorship. For example, in PO’s career, pioneering work in the medical Spanish field has paved the path for language equity as a topic of growing scholarly inquiry in academic medicine, with more journals publishing related work [61] and some accepting curricular content in non-English languages [62]. Another strategy is to engage with scholars outside of medicine who may be working on related topics. For example, scholars in the humanities, applied linguistics, public health, and health disciplines outside of medicine may all conduct work related to medical language education. Cross-disciplinary collaborations can enhance the field and may also open the door to additional funding opportunities [63].

For some, the idea of self-promotion may be felt to be antithetical to personal, familial, or cultural guiding principles and may be considered overly ambitious, selfish, or aggressive [64]. Discomfort with self-promotion is more common among women and those who identify with minoritized racial/ethnic groups [64]. Some have proposed a dual promotion [65] or gratitude framework as ways to describe individual achievements as being made possible thanks to the support of others [45]. Using frameworks such as these, achievements can be viewed as opportunities to celebrate and lift up peers or the community, a concept that may alleviate some of the distress that people may experience when focusing on self-promotion.

## The Innovative Format Roadblock

*He had helped to design an interactive app to boost proficiency in diagnostic reasoning. It had started as a hobby and an outlet for fun, but, once learners got hold of it, it was clear that he was onto something. He did his best to explain how the app impacted the educational ecosystem in his personal statement and listed it within his curriculum vitae (CV) for promotion to associate professor*.

*Upon reading the submission, the promotion and tenure committee chair scratched her head in confusion and said, “I love using this on my smartphone. But this is way too ‘out there’ to put in as part of a promotion portfolio.”*

Innovative formats for scholarly outputs, like writing blogs, producing podcasts, engaging in graphic medicine, developing an app, or creating serious board games (games that have additional objectives besides having fun) may not fit within the traditional structures of promotion packets or CVs. Additionally, promotion criteria may be perceived as being rigid and unchangeable. Faculty who serve on promotion committees are often individuals who have themselves successfully navigated traditional criteria in their own career. Without an intentional plan to critically review and consider revisions to promotion criteria over time, committees may inadvertently perpetuate a confirmation bias that selects for the types of scholarship that have historically been considered acceptable while disincentivizing equally meritorious innovative work.

### Recommendations for Institutional Leaders to Incentivize Innovative Scholarship Formats

Academic institutions and leaders can incentivize innovative formats for scholarship by creating standardized approaches to track efforts that acknowledge new avenues for scholarship and impact. Medical education curricula and assessment practices should reflect content that aligns with population health needs, including social determinants of health and pedagogy that is responsive to the dynamic needs of learners and patients [66,67]. For example, Duke University School of Medicine’s promotion and tenure guidelines were revised in 2021 in recognition of the far-ranging interests, talents, and pursuits of faculty supporting the institution’s mission. As part of this revision, the Career Track was developed to recognize the various expressions of scholarship that faculty were pursuing [68]. Also, additional rubrics were developed to support the evaluation of productivity and impact in nontraditional formats (e.g., digital media and advocacy) [69]. MLB participated in the application of these new criteria in the Department of Pediatrics, and she continues to do so across the School of Medicine. A plan for revisiting and revising promotion criteria on a periodic basis may help capture new scholarly trends, topics with timely social impact, technological advances, and platforms for dissemination as they evolve over time. Some scholars have described successful ways to track and define the impact of new forms of scholarship, such as tweetorials [70,71,72] or graphic medicine [73,74].

### Recommendations for Scholars Using Innovative Scholarship Formats

It is essential that individuals in academic medicine understand how to showcase their scholarly achievements in a way that maximizes their chances of career advancement. One way to navigate this is to conduct traditional scholarship about said innovation, a concept that can be framed as a form of *metascholarship*: Applying traditional research methodologies, such as conducting scoping or narrative reviews, evaluating pedagogical effectiveness, and collecting qualitative data through focus groups or semi-structured interviews, may be an important step to get innovative formats recognized within the bounds of traditional publication venues and conference presentations. For instance, beyond *creating* a serious board game, TMC led a research program to examine the outcomes and teaching potential of the game [75,76,77,78]. Similar work has been done to evaluate the use of graphic medicine as a pedagogic tool [79] and the impact of an open access blog or podcast [80,81,82].

Scholars whose work products do not fit into any existing section of institutionally structured CVs may still consider including them by creating a novel CV heading, such as “Additional forms of scholarship” or “Additional scholarly products.” Scholars may also choose to develop their own headings to best capture their specific innovation category, such as “Digital publications,” “Non-print media,” “Scholarly podcasts,” “Graphic medicine,” or “Visual media.” If permitted, the CV should be accompanied by an appendix or supplemental portfolio with relevant work samples of the innovative work such as images, transcripts, or links for accessing materials. These strategies are important for tracking the work’s impact and setting a precedent by exposing promotion committees and senior leadership to viable approaches for documenting innovative scholarly products.

## Conclusion: Clearing Traditional Roadblocks

**Figure 1:**
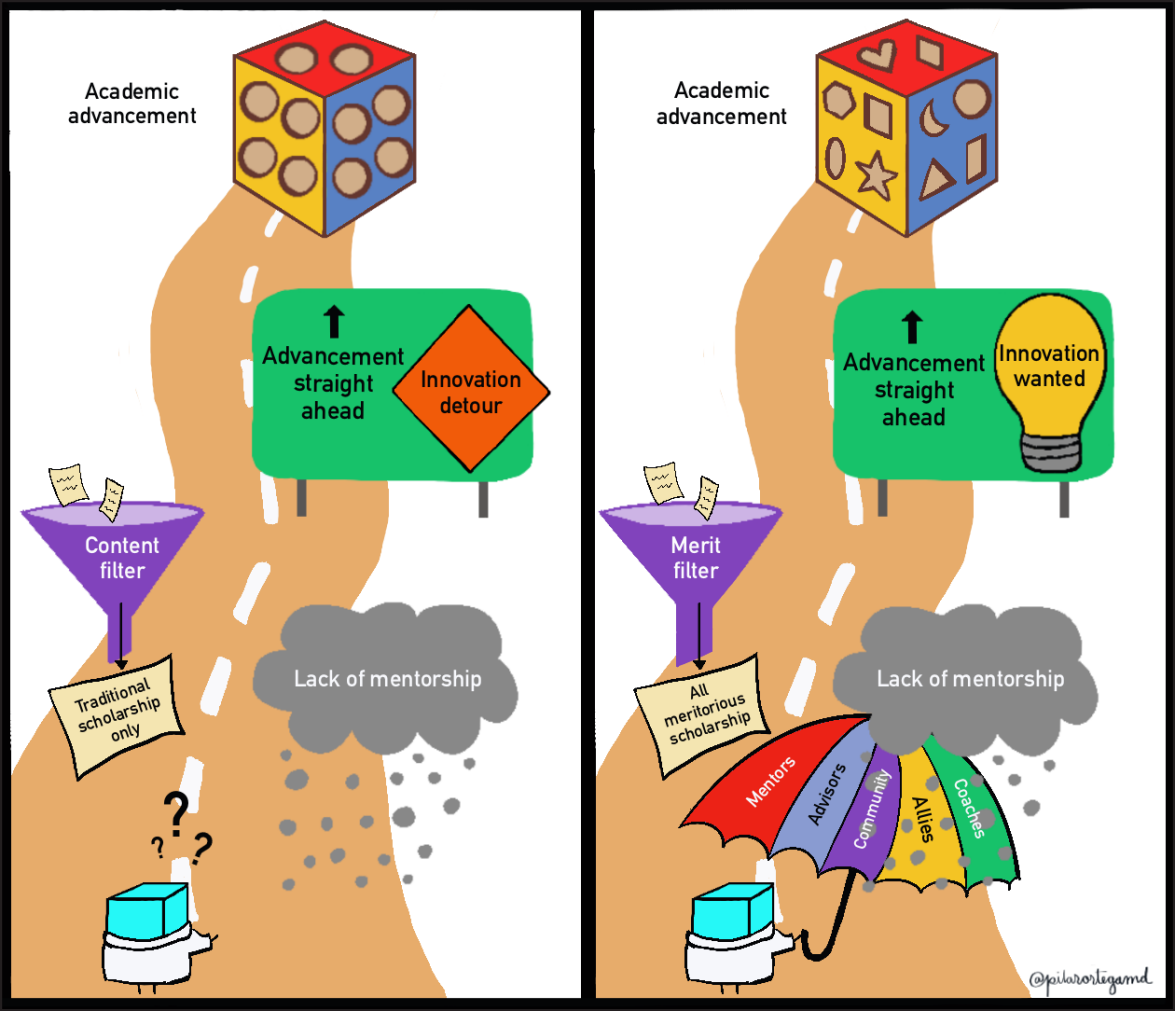
**Facing Traditional Roadblocks in Academic Medicine Can Feel Like Being a Square Peg in a White Coat**. The academic medicine pathway is lined with traditional roadblocks to achieving academic advancement for those with atypical journeys, innovative scholarship formats, or nontraditional scholarly interests (left side of the illustration). Strategies to clear roadblocks and enhance nontraditional scholar retention and advancement in academic medicine include providing mentorship, acknowledging population health and learner needs as key aspects of scholarly merit, and creating space for innovative scholarship formats (right side of the illustration).

Far from being an exhaustive list of roadblocks to the advancement for some members of the academic medicine community, our stories represent examples of big and small ways in which individuals are sometimes excluded from reaching their full potential and contributing to the positive growth of academic medicine by virtue of being considered nontraditional (Figure 1).

The term nontraditional is vague and singles out scholars for not fitting in with traditional expectations. Instead, it may be more productive to develop specific strategies to enable scholars who use innovative formats, focus on nontraditional topics, or have atypical journeys in medicine to appropriately demonstrate the merit of their accomplishments. As we respond to the evolving needs of patients, learners and faculty, pathways to advancement in academic medicine must be inclusive, dynamic, and innovative.
